# Exploratory Study on Microbiota and Immune Responses to Short-Term *L. paracasei* CNCM I-1518 Consumption in Healthy Adults

**DOI:** 10.3390/nu17142287

**Published:** 2025-07-10

**Authors:** Fernando Rivero-Pino, Maria José Castro, Paz Redondo del Río, Eloina Gutierrez, Agustín Mayo-Iscar, Mercedes Nocito, Alfredo Corell

**Affiliations:** 1 Department of Medical Biochemistry, Molecular Biology, and Immunology, School of Medicine, University of Seville, 41009 Seville, Spain; 2 Instituto de Biomedicina de Sevilla, IBiS, Hospital Universitario Virgen del Rocío, CSIC, University of Seville, 41013 Seville, Spain; 3 Departamento de Enfermería, Universidad de Valladolid, 47003 Valladolid, Spain; 4 Departamento de Pediatría, Inmunología, Obstetricia-Ginecología, Nutrición-Bromatología, Universidad de Valladolid, 47005 Valladolid, Spain; paz.redondo@uva.es (P.R.d.R.); elogut@gmail.com (E.G.); 5 Departamento de Estadística e Investigación Operativa & IMUVA, Universidad de Valladolid, 47011 Valladolid, Spain; agustin.mayo.iscar@uva.es; 6 Inmunología, Hospital Clínico de Zaragoza, 50009 Zaragoza, Spain; mnocitocolon@gmail.com

**Keywords:** *L. paracasei*, Actimel, gut microbiota, immunomodulation

## Abstract

Background/Objectives: The gut microbiota and immune system are interconnected, with targeted nutritional interventions offering potential to modulate immune function. This study aimed to evaluate the short-term immunomodulatory effects of *Lacticaseibacillus paracasei subspecies paracasei* CNCM I-1518 (*L. paracasei* CNCM I-1518) in healthy adults. Methods: A 15-day dietary intervention was conducted involving healthy adults. Nutritional status, dietary habits, and systemic immune biomarkers were assessed, alongside changes in gut microbiota composition. Results: The results revealed significant effects on both cellular and humoral immunity. Cellular immunity was enhanced through increased circulating B lymphocytes, absolute monocyte counts, and leukocyte numbers, alongside reduced eosinophil levels, potentially mitigating allergic responses. Humoral immunity was improved by elevated serum IgG1, IgG2, and IgG4 levels, enhancing defenses against pathogenic antigens, and increased serum complement proteins C3 and C4, supporting innate immunity. Microbiota analysis showed a reduction in *Clostridium* and the *Clostridium/Escherichia coli* ratio, with a notable increase in the *Lactobacillus/Clostridium* ratio, highlighting the strain’s ability to reshape intestinal bacterial balance. Conclusions: A short-term intake of *L. paracasei* CNCM I-1518 can simultaneously modulate immune function and gut microbiota composition, supporting its potential as a targeted dietary intervention to promote immune health.

## 1. Introduction

The immune system serves as the body’s main defense mechanism, managing complex responses against pathogens while maintaining tolerance towards harmless antigens and host tissues [1]. The gut, a central site of immune activity, the place where a foreign substance is either “tolerated” or considered an “enemy”, is also home to an extensive and diverse microbial community that interacts continuously with the host’s immune cells. These interactions play a critical role in the immune responses’ modulation to ensure effective protection. In this context, probiotics, defined as live microorganisms that provide health benefits to the host when consumed in adequate amounts [2], have been investigated for their capacity to modulate the immune system and enhance overall health outcomes [3].

The intake of probiotics, which often include strains from genera such as *Lactobacillus* (LAB) or *Bifidobacterium*, has been linked to modifications in the gut microbiota composition and activity [3,4]. These bacteria can alter the gut microbiota composition by producing metabolites such as lactic acid and short-chain fatty acids that lower intestinal pH, creating an environment unfavorable to pathogenic bacteria. They also compete for nutrients and adhesion sites on the gut lining, limiting colonization by harmful microbes and promoting the growth of beneficial microbial populations. This, in turn, may influence the host’s immune functions through different mechanisms. Probiotics may reinforce the gut barrier integrity, exert antagonism that prevents pathogens from multiplying, be responsible for the production of toxins that prevent the pathogenic action of these agents, and directly interact with immune cells to regulate local and systemic immune responses [2,5]. These effects could lead to enhanced production of immunoglobulins, activation of natural killer (NK) cells, and modulation of cytokine profiles. Despite increasing evidence suggesting that probiotics can exert positive immunomodulatory effects, their impact appears to be highly strain-specific, influenced by factors such as dosage, duration of intake, and the host’s health status [6,7].

While the potential of probiotics to influence immune function has been widely reported, existing studies present conflicting results due to variability in study design, strain selection, and population characteristics [8]. There is a need for more standardized and comprehensive research to clarify these effects, as the inconsistent findings have limited the development of clear clinical guidelines for probiotic use in immune support. Nevertheless, several clinical trials have demonstrated that probiotics may reduce the risk of infections, alleviate symptoms of immune-related disorders, and support overall immune function, particularly in vulnerable populations such as the elderly, children, individuals with chronic diseases, individuals with travel diarrhea or rotavirus, and those under immunosuppressive therapies [9].

For probiotics to influence the immune response, it is essential that they survive after passing through the gastrointestinal tract in order to express their immunomodulatory properties. Lactic acid bacteria are able to colonize the intestine temporarily and survive during intestinal transit. Furthermore, due to their transient adhesion to the epithelium or the transepithelial sensing of immune cells, they can modify the local immune response of the host. In this regard, it has been observed that certain strains of LAB have effects on delayed hypersensitivity reactions, antibody production, and functional activation of macrophages [6,7]. On the other hand, it has also been shown that some strains are capable of preventing enteric infections, as well as exerting an antitumor action by inhibiting carcinogenic chemical agents. Specifically, the immunomodulatory properties of lactic acid bacteria in humans have been described several times by different groups of researchers. In general, it has been observed that the ingestion of lactic bacteria is capable of producing an increase in the number of immunoglobulin-producing cells and the secretion of specific antibodies in animals subjected to viral infections [10,11]. A systematic review and meta-analysis have shown that the consumption of fermented dairy drinks containing *L. casei* twice a day may reduce the incidence of common infectious diseases. However, it also highlighted the need for standardization of clinical outcomes related to immune function and guidance on confounding factors to be considered [12].

Based on the increasing recognition of the gut microbiota’s role in modulating the immune system at both the mucosal and systemic levels, this study aims to explore how targeted nutritional interventions can influence this intricate relationship. Specifically, this research aimed to evaluate the in vivo immunomodulatory effects of a probiotic strain from the *Lactobacillus* genus in a sub-acute intervention with healthy adult participants. By assessing the nutritional status of participants, monitoring their dietary habits, and implementing specific dietary interventions, this study was designed to explore changes in gut microbiota composition and their subsequent impact on systemic immune parameters. Ultimately, this work aims to enhance our understanding of the potential benefits of probiotic supplementation in supporting immune health through modulation of the gut microbiota.

## 2. Materials and Methods

### 2.1. Study Population and Dietary Assessment

The study included 21 adult participants, aged 19–46 years (19 female participants and 2 male participants, clinical). No a priori power analysis was conducted to determine the sample size. The selection criteria included the following parameters: body mass index (BMI), calculated as weight (kg) divided by height squared (m^2^), between 18.5 and 29.99; waist-to-hip ratio with thresholds set at <0.90 for males and <0.85 for females; and the percentage of body fat: considered within the normal range if <30%. Exclusion criteria included any diagnosed chronic or acute illnesses, use of medications or dietary supplements that could affect immune or metabolic function, recent infections (within 4 weeks), or any condition that, in the opinion of the investigators, could interfere with the interpretation of the study data.

Note that the sex distribution of participants (19 females, 2 males) reflects the actual composition of the enrolled students in the topic “Immunonutrition” at the Human Nutrition and Dietetics program at the University of Valladolid, from which the study sample was recruited. Standard biochemical and anthropometric measurements were carried out on all the subjects. The study was conducted according to the Good Practice Guidelines and in line with the principles outlined in the Declaration of Helsinki of the World Medical Association. This study (code PROBIOINMUNE) was approved by the Clinical Research Ethics Committee of the University of Valladolid on 14 October 2013, and written informed consent was obtained from all participants before entering the study. This study is registered in Clinical Trials (NCT06747143).

### 2.2. Nutritional Intervention

The intervention followed a sequential design, where the same group of 21 participants were subjected to the different dietary interventions sequentially (phases). Each phase lasted 15 days, without a wash-out period, and participants were evaluated before and after each intervention phase, allowing for a direct comparison of the effects of the different dietary treatments. The interventions were as follows:

Phase 1 (Baseline): participants followed their regular diet without any restrictions.

Phase 2 (Fermented animal-based foods-free): participants continued their regular diet but completely excluded all dairy products containing live microorganisms (e.g., fermented milk, yogurt, certain sausages, and cheeses).

Phase 3 (Probiotic Supplementation): Participants followed their regular diet supplemented with two daily servings (100 mL × 2) of a probiotic product containing *Lacticaseibacillus paracasei subspecies paracasei* CNCM I-1518 (*L. paracasei* CNCM I-1518, previously known as *Lactobacillus casei* DN-114001). It has to be noted that *L. paracasei* CNCM I-1518 is present alive in Actimel^®^ (DANONE, Paris, France) in levels of 10^8^ CFU/g or 10^10^ CFU/100 g bottle, and several studies have confirmed the survival of the strain through the intestinal tract at levels consistent with the ability to provide a beneficial physiological effect [13]. Participants were instructed to take the probiotics 30 min before the meals.

At the end of each phase (day 15), blood, saliva, and fecal samples were collected for analysis. Considering this study design, participants were analyzed at three time points, with each individual serving as their own control. The impact of eliminating products containing live microorganisms on the immune system was evaluated, along with the immunomodulatory effects of probiotic supplementation. Dietary assessment was performed using a 10-day non-consecutive prospective food diary, which included three weekend days to account for variability in intake. Participants recorded their dietary intake on one day five days prior to the beginning of Phase 1 and on three separate days during each intervention phase (specifically on days 5, 9, and 12 of each 15-day period). Macronutrient and micronutrient intake was analyzed using the “Alimentación y Salud” software developed by Mataix-Verdú J [14]. Ref. [14] Participants recorded their own dietary habits using structured food diaries, and nutrient intake was subsequently calculated by qualified nutritionists based on these records.

### 2.3. Biochemistry and Hematology

General biochemistry and hematology were performed after each phase (three blood extractions). Biochemical analysis was carried out using the Sysmex XE-2100 (Roche, Basel, Switzerland), while hematological determinations were performed using the Hitachi Modular DPP (Roche, Basel, Switzerland). Reference or normality values are those as defined by the Biochemistry and Hematology Laboratory of the University Clinical Hospital of Valladolid.

### 2.4. Measurement of Immune System Function Markers

All participants underwent a complete blood count (CBC) to determine the absolute number of circulating leukocytes and lymphocytes, as described elsewhere [15].

#### 2.4.1. Immunochemistry

In relation to humoral immunity, serum immunoglobulins G, A, M, and G subclasses, as well as the complement C3 and C4 factors, were determined. All these parameters were determined using the Dade Behring BNII Nephelometer [16] from Siemens (Munich, Germany) [15].

#### 2.4.2. Cellular Immunity

For the analysis of lymphocyte subpopulations, flow cytometry analysis was performed using a FACScalibur flow cytometer (Becton Dickinson^®^, Franklin Lakes, NJ, USA) and direct immunofluorescence with a four-color technique. The following markers were assessed: (i) T Lymphocytes: CD3, CD4, CD8, CD45 RA, and CD45 RO (distinguishing between helper T cells, cytotoxic T cells, and naïve or memory subsets within each group). (ii) B Lymphocytes: CD19; (iii) NK Cells: CD16 and CD56. The laboratory protocol involved pipetting 50 µL of whole blood with EDTA anticoagulant into three 5 mL tubes [15]. Monoclonal antibody mixes were added as follows:

A total of 5 µL of CD3/CD4/CD8/CD45 in the first tube, 5 µL of CD3/CD56/CD19/CD45 + 5 µL of CD16 in the second tube, and 5 µL of CD45RA/CD45RO/CD3/CD4 in the third tube (all monoclonal antibodies from Becton Dickinson^®^). After vortexing and incubating in the dark at room temperature for 15 min, 500 µL of FACS LYSING was added, followed by another vortex and incubation for an additional 15 min. The percentage of each lymphocyte subset in the blood was then determined by flow cytometry [17].

### 2.5. Gut Microbiota Analyses

To assess the impact of probiotic consumption on gut microbiota, fecal samples were collected at each phase of the study and cultured for different microorganisms. Stool samples were collected from 12 participants (morning samples using sterile swabs) and subjected to serial dilutions in anaerobic hoods. Aliquots (50 µL) of each dilution (10^7^–10^9^) were plated on selective media: MacConkey agar for enterobacteria and *Escherichia coli*, blood agar for *Clostridium* species, and MRS agar for *Lactobacillus*. Plates were incubated at 37 °C for 4 days under strict anaerobic conditions with a gas phase of N_2_-CO_2_-H_2_ (88:5:7, vol/vol/vol). Colony-forming units (CFUs) were counted in duplicate for each anaerobe. Given that representative stool samples were used rather than total feces, the counts obtained are relative rather than absolute.

### 2.6. Data Analysis

First, the Kolmogorov test was performed on each set of results obtained in the trials to check that the data were normally distributed (symmetrical distribution), which was the case for all studied variables. Correlations between the variables showing significant changes between measurements were assessed to explain the observed variability during the nutritional intervention. Both parametric models (regression) and non-parametric curve estimation models were applied. These estimations were complemented with graphical representations to visualize the relationships found. Statistical analyses and model estimations were conducted using SPSS 22.0.

## 3. Results

### 3.1. Biochemistry and Hematology

Twenty-one adults (19 females, 2 males; aged 19–46 years) participated in the study. Participants were recruited from the “Immunonutrition” course at the University of Valladolid, reflecting the class’s sex distribution. Inclusion criteria included a BMI of 18.5–29.99 kg/m^2^, a waist-to-hip ratio of <0.90 (males) or <0.85 (females), and body fat <30%. Standard anthropometric and biochemical assessments were performed. The study followed a sequential intervention design with 15-day phases, no wash-out period, and evaluations before and after each phase. Ethical approval was obtained, and informed consent was provided.

Biochemical (Supplementary Material—Table S1) and hematological (Supplementary Material—Table S2) parameters are presented as average ± standard deviation (values between brackets correspond to the minimum and maximum value quantified in the whole population). In the table, statistically significant changes identified are reported in the table as footnotes, indicating which groups compared led to that change. As it can be observed, significant differences observed between different time points (baseline or extraction 1, deprivation of lactic acid bacteria or extraction 2, and supplementation with lactic acid bacteria or extraction 3) were reported, although in general, values always remained within normal physiological levels. Reference or normality values are those as defined by the Biochemistry and Hematology Laboratory of the University Clinical Hospital of Valladolid.

### 3.2. Lymphocyte Subpopulations

Lymphocyte subpopulations (Table 1) are presented as average ± standard deviation (values between brackets correspond to the minimum and maximum value quantified in the whole population). In the table, statistically significant changes identified are reported in the table as footnotes, indicating which groups compared led to that change. As it can be observed, significant differences observed between different time points (baseline or extraction 1, deprivation of lactic acid bacteria or extraction 2, and supplementation with lactic acid bacteria or extraction 3) were found.

### 3.3. Immunochemistry

In Table 2, the results obtained for the immunochemistry are reported, including the changes observed in the serum immune proteins after each phase of the study. Values are presented as average ± standard deviation (values between brackets correspond to the minimum and maximum value quantified in the whole population). In the table, statistically significant changes identified are reported in the table as footnotes, indicating which groups compared led to that change.

### 3.4. Gut Microbiota Analyses

In Table 3, the results obtained from the fecal flora analyses during the three phases of the study are reported. Values are presented as average ± standard deviation of the relative values in logarithmic base (values between brackets correspond to the minimum and maximum value quantified in the whole population). In the table, statistically significant changes identified are reported in the table as footnotes, indicating which groups compared led to that change.

### 3.5. Correlation Among Variables

**Figure 1:**
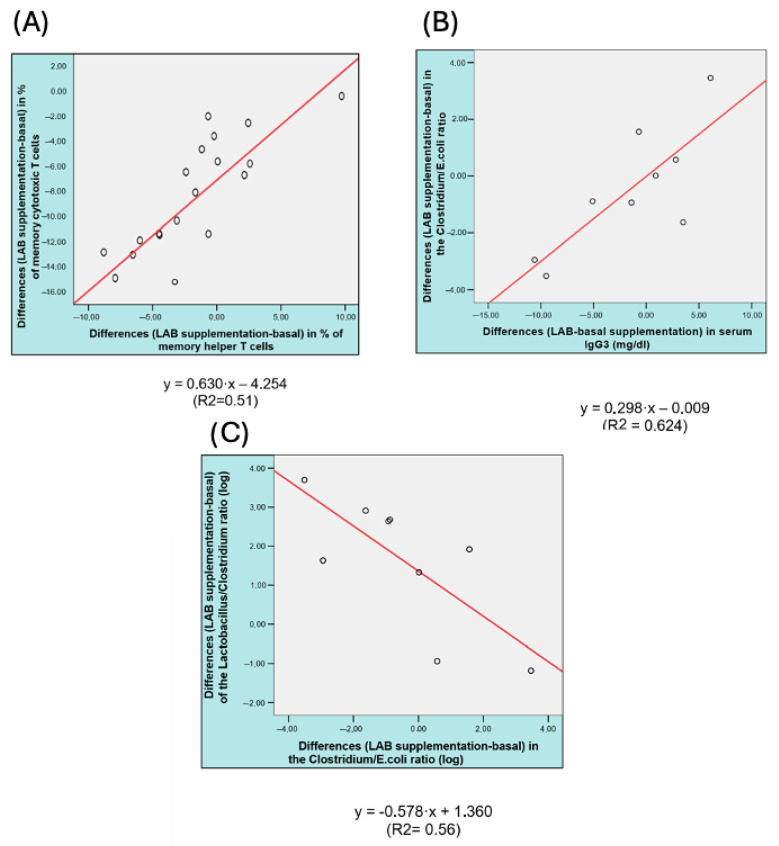
Correlation graphics (difference LAB supplementation—basal) of (**A**) % of ratio of memory cytotoxic T lymphocytes vs. % of memory helper T lymphocytes (*p* < 0.001); (**B**) ratio of *Clostridium/E. coli* vs. IgG3 (*p* < 0.01); and (**C**) *Lactobacillus/Clostridium* vs. *Clostridium/E. coli* (*p* < 0.02).

The potential correlation among different parameters was evaluated. The analysis was conducted using differences in variables between different time points that were previously identified as significant through regression methods, and the most relevant are shown in Figure 1 (all tested correlations are shown in Supplementary Table S3).

## 4. Discussion

### 4.1. Biochemistry and Hematology

Significant changes (*p* < 0.05) were observed in certain biochemical variables; however, overall values remained largely within or near the normal physiological range, indicating no severe biochemical dysregulation.

These changes might not be considered biologically relevant as the values still fall within physiological levels, but there are indications that the intake of the probiotic could have an impact on the leukocyte’s levels, as observed in other studies carried out on healthy subjects [18]. The percentage of red cell distribution width (RDW) decreased significantly during LAB deprivation and subsequently increased beyond baseline levels following a 15-day supplementation with *L. paracasei* CNCM I-1518 (*p* < 0.01). RDW values shifted from 13.83% at baseline to 13.63% during deprivation and rose to 14.57% after supplementation. In this line, red cell distribution width has been identified as a strong predictor of *Lactobacillus* sp. composition in the adult population, showing a positive association with the presence of *L. helveticus* and the absence of *L. ruminis* [19]. In this regard, different results have been published. For instance, it has been reported by a randomized, double-blind, controlled trial in 96 healthy adults examining the safety (primary endpoint) and gut microbiota response (secondary endpoint) to a multi-strain fermented milk product containing *Lactobacillus paracasei* CNCM I-1518, CNCM I-3689, and *Lactobacillus rhamnosus* CNCM I-3690. Daily ingestion of one or three bottles for four weeks showed no significant safety differences compared to the control across adverse events, vital signs, metabolic and inflammatory markers, and digestive symptoms. Probiotic strains were detected only during consumption, with higher levels in the three-bottle group. Gut microbiota diversity remained unchanged, though a few bacterial genera showed dose-dependent differences [20]. However, the study included three strains, whereas the study hereby described focuses on the impact of one specific strain.

In spite of the observed hematological changes, the physiological levels were maintained, and this variation is consequently not associated with specific benefits following the consumption of the strain for 15 days. The findings align with observed changes in innate cellular immunity (discussed in the next sections), including an increase in circulating monocytes and a decrease in eosinophils following the ingestion of *L. paracasei* CNCM I-1518.

### 4.2. Innate Immunity

#### Humoral Immunity

The complement system represents the most important humoral effector mechanism of the innate immune response. Its primary functions include defending the host against infections by microorganisms, initiating the inflammatory response, and clearing circulating immune complexes from the bloodstream. The complement system comprises a cascade of proteins that act sequentially to eliminate pathogens or their toxins while inducing inflammation. Among these proteins, C3 is the most abundant in the bloodstream [21].

In this study, serum quantification of complement proteins C3 and C4 at three distinct time points revealed significant variations. A marked decrease in serum C3 and C4 levels was observed when LAB were removed (107.94 mg/dL vs. 94.78 mg/dL for C3 and 19.49 mg/dL vs. 16.40 mg/dL for C4, *p* < 0.01). However, supplementation with *L. paracasei* CNCM I-1518 significantly increased the levels of both proteins (94.78 mg/dL vs. 104.09 mg/dL for C3 and 16.40 mg/dL vs. 18.09 mg/dL for C4, *p* < 0.05). The observed increase in C3 and C4 levels following *L. paracasei* CNCM I-1518 ingestion is expected to enhance the activation of their downstream products, C3a and C4a, which function as anaphylatoxins and potentiate the inflammatory response. This could also be relevant, considering that it has been recently reported that low serum concentrations of C3 and C4 have been significantly associated with the presence of severe disease and increased mortality in patients with COVID-19 [22].

These findings suggest the correlation between C3 and C4, increasing following a similar trend, in promoting immune functions such as pathogen clearance and inflammation.

### 4.3. Cellular Immunity

Significant variations in absolute monocyte counts (cells/μL) were observed during the study. Monocyte levels decreased when LAB were removed from the diet. In contrast, supplementation with *L. paracasei* CNCM I-1518 led to a significant increase in absolute monocyte numbers (*p* < 0.01) compared to the LAB-deprivation phase. Monocytes, as antigen-presenting and phagocytic cells, play a central role in the immune response. Their increase following supplementation with *L. paracasei* CNCM I-1518 suggests an enhancement of these immune functions. Similarly, leukocyte counts in the blood increased significantly relative to baseline following supplementation with the test item. This elevation indicates an overall stimulation of innate immunity associated with probiotic supplementation [23].

On the other hand, eosinophils, which are key players in pro-inflammatory processes and the pathogenesis of allergic diseases, including immediate hypersensitivity and parasite death, exhibited a significant decrease in percentage (*p* < 0.05) when *L. paracasei* CNCM I-1518 was included in the diet. This reduction implies a potential benefit of the supplementation in mitigating allergic conditions by reducing eosinophil-mediated inflammation [24].

However, other studies have shown different results. In this regard, the effect of a probiotic supplementation on immune and hormonal changes during high-intensity training was evaluated, and for total and differential leukocyte and lymphocyte subsets, no statistical differences were recorded between the groups (test item and placebo), in spite of an observed greater increase in leukocytes, neutrophils, and CD19+ concentrations in the control group at the end of training [25]. Guillermard et al. [10], on the other hand, reported in a randomized, double-blind, controlled-in-two-parallel-groups, monocentric nutritional intervention that the consumption of the test item (*L. casei*) for 3 months led to a statistically significant higher increase in leukocytes and in NK cell absolute counts, but no changes for other immune parameters evaluated.

Regarding the observed results in immunoglobulins and eosinophils, it is important to highlight that these changes, although modest, could have clinically relevant implications, particularly in individuals with increased susceptibility to respiratory infections or with a history of allergic diseases [26]. These preliminary results require further investigation.

### 4.4. Adaptive Immunity

#### Humoral Immunity

Regarding humoral immunity, immunoglobulins, also known as antibodies, are glycoproteins found in plasma and tissues. They function as antigen receptors on B lymphocytes.

A significant increase (*p* < 0.01) in IgG1, IgG2, and IgG4 levels was observed during the ingestion of *L. paracasei* CNCM I-1518 compared to both baseline and the condition of lactic acid bacteria deprivation. Studies in humans have shown that the production of IgG subclasses varies depending on the antigen that triggers the immune response. Polysaccharide antigens predominantly induce IgG1 and IgG2 synthesis, while viral antigens mainly stimulate IgG3 and IgG4 production [27]. For IgG3, a significant change (*p* < 0.05) was detected only between the baseline and deprivation conditions, indicating reduced complement activation when no lactic acid bacteria are consumed. Although an increase in IgG3 levels was noted with supplementation, this change was not statistically significant. No significant changes were observed in other immunoglobulins, including IgA, total IgG, and IgM.

These findings suggest that the ingestion of *L. paracasei* CNCM I-1518 for 15 days potentially enhances activity against bacterial antigens, as evidenced by increased serum levels of IgG1 and IgG2. Long-term studies are needed in order to evaluate if other changes occur and to confirm that the observed changes are maintained.

### 4.5. Cellular Immunity

A significant decrease (*p* < 0.01) in the population of double-positive CD3+ CD4+ CD8+ cells was observed between diets with deprivation of lactic acid bacteria and those supplemented with *L. paracasei* CNCM I-1518. These results suggest that the full microbial diversity of fermented foods may be necessary to maintain this T-cell subset and that single-strain supplementation is not sufficient to replicate this effect.

The percentage of double-negative CD3+ CD4− CD8− cells, an indirect marker of most Tγδ lymphocytes, significantly decreased (*p* < 0.0000001) when lactic acid bacteria were excluded from the diet. These percentages increased upon supplementation with *L. paracasei* CNCM I-1518, although the increase was not statistically significant. In the same line, natural killer (NK) cell percentages significantly decreased (*p* < 0.05) compared to baseline levels upon removal of lactic acid bacteria from the diet. A 15-day supplementation with *L. paracasei* CNCM I-1518 was insufficient to restore baseline percentages. Current data are insufficient to determine a correlation between probiotic supplementation and enhancement of NK cell function, given the large heterogeneity in the studies [28].

A significant reduction (*p* < 0.01) in CD3+ CD56+ cells (CD8+ T lymphocytes with HLA-unrestricted cytotoxicity) was observed when comparing baseline diets with diets supplemented with *L. paracasei* CNCM I-1518. CD19+ B cell percentages increased significantly (*p* < 0.01) following dietary supplementation with the test item compared to baseline levels. This increase of B lymphocytes has been reported for other strains of *Lactobacillus*. For example, *Lactobacillus rhamnosus* orally administered at a dose of 10^7^ cfu/10 μL every other day for 2 weeks was shown to promote the development and maturation of B lymphocytes, enhance the activation and antigen-presentation ability of B lymphocytes, and regulate the secretion of immunoglobulin by B lymphocytes in mice [29]. However, as indicated previously, the effects of specific strains might not be extrapolated to others, and animal studies should not be interpreted as evidence for humans. In this regard, it has also been reported that, in 41 healthy sedentary males, probiotic (*L. acidophilus*, *L. lactis*, *L. casei*, *B. longum*, *B. bifidum*, and *B. infantis*) consumption for 12 weeks had no effect on B-lymphocyte cell count [30]. As reviewed recently, studies conducted on animal models suggest the potential of prebiotics, probiotics, and synbiotics in managing or preventing diseases related to B-cell immunoregulation. Nonetheless, these findings must be confirmed in humans, emphasizing a thorough analysis of B-cell subpopulations rather than limiting the assessment to the humoral immune response [31].

Among T cells, naïve T lymphocytes significantly increased (*p* < 0.01) following the removal of lactic acid bacteria from the diet and subsequently decreased significantly (*p* < 0.01) upon supplementation with *L. paracasei* CNCM I-1518. The fluorescence intensity of CD45RA, expressed by these lymphocytes, significantly decreased when the diet was supplemented with the test item. A significant increase (*p* < 0.05) in memory T lymphocytes was observed following the removal of lactic acid bacteria from the diet (44.28% vs. 42.19%). This percentage significantly decreased (*p* < 0.05) upon supplementation with *L. paracasei* CNCM I-1518 (44.28% vs. 41.08%). The intensity of CD45R0 expression on these cells increased in the absence of lactic acid bacteria and decreased again following supplementation with *Lactobacillus casei* (*p* < 0.001). It has been reported that the consumption of milk fermented with yogurt cultures plus *Lactobacillus casei* DN-114001 by students under academic examination stress increased absolute lymphocyte and CD56 cell counts compared to the control group, suggesting immunomodulatory benefits in stressful conditions [32].

For naïve CD4+ helper T lymphocytes, a significant decrease (*p* < 0.05) was observed when the diet was supplemented with the test item compared to the deprivation phase, with a similar decrease in fluorescence intensity. Memory helper T lymphocytes also showed a slight but significant decrease (*p* < 0.05) upon supplementation with *L. paracasei* CNCM I-1518 compared to the deprivation phase (30.27% vs. 28.41%). Their fluorescence intensity significantly increased in the absence of lactic acid bacteria and decreased following supplementation. In this regard, Meyer et al. [33] reported, in a group of 33 young healthy women (22–29 years) who consumed 100 g/day of either probiotic or conventional commercially available yogurt for 2 weeks and 200 g/day for another 2 weeks, followed by a 2-week wash-out period with no fermented food at all, that in the probiotic group only, the numbers of cytotoxic T lymphocytes (CD3+ CD16+ CD56+) increased significantly, while there were no major changes for other cell populations, and all remained within the physiological range. Also, Ortiz-Andrelluchi et al. [34] reported that 6 weeks of probiotic intake by women (randomized, controlled, and double-blind nutritional intervention study with parallel groups) led to a non-significant increase in T and B lymphocytes and a significant increase in natural killer cells. Naïve cytotoxic T lymphocytes (CD8+) significantly decreased following supplementation with *L. paracasei* CNCM I-1518 compared to the deprivation phase (21.13% vs. 19.35%, *p* < 0.05), with a concurrent significant decrease in fluorescence intensity. Similarly, memory cytotoxic T lymphocytes significantly decreased upon supplementation (12.78% vs. 11.18%, *p* < 0.05). The fluorescence intensity of these cells significantly increased in the absence of lactic acid bacteria and decreased following supplementation.

The most informative parameter for evaluating T cell subpopulations was the naïve/memory helper T cell ratio. This ratio decreased following supplementation with *L. paracasei* CNCM I-1518, indicating a relative increase in memory or activated helper T lymphocytes. This decrease was attributable to a greater reduction in naïve T lymphocytes compared to memory T lymphocytes. Overall, a reduction in the percentages of naïve and memory helper T lymphocytes was observed following supplementation with the test item. The naïve/memory helper T cell ratio also decreased during supplementation, reflecting a more pronounced reduction in naïve T cells. Similarly, naïve and memory cytotoxic T lymphocytes showed a significant reduction following supplementation. These changes suggest that the reduction in naïve and memory T lymphocytes affects all T cell subpopulations analyzed.

The observed changes in T cell subpopulations, specifically the decrease in the naïve/memory helper T cell ratio following supplementation with *L. paracasei* CNCM I-1518, suggest a shift toward a more mature or activated T cell profile. Probiotics, particularly strains of *Lactobacillus*, are known to modulate T cell dynamics by enhancing immune responses or promoting tolerance depending on the context [35,36]. Further studies should explore whether these shifts persist long-term and their implications for immune-mediated diseases, particularly in the context of chronic inflammation or infection [37].

In Figure 1A, the observed linear relationship between the change in the percentage of cytotoxic/memory T cells (*Y*-axis) and the percentage of T-helper/memory cells (*X*-axis) following probiotic supplementation suggests a coordinated immune modulation effect. A total of 51% of the variance in the change of cytotoxic/memory T cells can be explained by the variation in T-helper/memory cells, suggesting a moderately strong association. This finding supports the hypothesis that probiotic supplementation can influence adaptive immune responses, particularly through its effects on memory T cell populations. An increase in T-helper/memory cells is associated with a proportional increase in cytotoxic/memory T cells. This alignment may reflect the role of T-helper cells in providing support to cytotoxic cells via cytokine secretion or direct cellular interactions, facilitating enhanced cytotoxic memory responses.

Regarding total T lymphocytes (CD3+), helper T lymphocytes (CD3+ CD4+), and cytotoxic/regulatory T lymphocytes (CD3+ CD8+), no significant changes were observed throughout the study. Additionally, there were no significant changes in the CD4/CD8 ratio, a key nutritional index. The T/B lymphocyte ratio (CD3+/CD19+) decreased in the absence of lactic acid bacteria and further decreased following supplementation with the test item. This reduction was attributable to an increase in B cell percentages in both conditions, while the percentage of total T lymphocytes remained unchanged. This suggests that this microorganism selectively modulates the immune response by promoting B cell proliferation, potentially enhancing humoral immunity, while maintaining stable T lymphocyte levels, highlighting its role in shaping specific immune cell dynamics.

### 4.6. Fecal Flora Analyses

Considering the rapid modulation of gut flora by medications and dietary products, a 15-day period in each phase was deemed sufficient to observe effects on fecal microbiota [38]. A significant reduction in *Clostridium* levels (*p* < 0.05) was observed when patients consumed *L. paracasei* CNCM I-1518 compared to the baseline diet. Conversely, a significant increase (*p* < 0.05) in the *Clostridium/E. coli* ratio was noted during the LAB-deprivation phase compared to baseline, indicating a rise in *Clostridium* levels in feces when no lactic acid bacteria were consumed. No significant differences were found in the *Lactobacillus* count, different from what has been reported in other studies [13], where the consumption by 12 healthy subjects after 10 days of a fermented milk containing *Lactobacillus casei* DN-114001 was accompanied by a high, transient increase in the quantity of this strain in the feces of all of the subjects without markedly affecting biochemical or bacteriological factors. Recently, Fehlbaum et al. [39] showed that daily addition of *L. paracasei* induced minor microbiota changes, increasing *Lactobacillus*, *Enterococcus*, and *Faecalibacterium*, and modulating gene expression, particularly in *Faecalibacterium*, with enhanced carbohydrate utilization.

However, it must be noted that the fecal *Lactobacillus/Clostridium* ratio showed a significant increase (*p* < 0.05) when comparing the baseline diet with the diet supplemented with *L. paracasei* CNCM I-1518. This observed increase in the *Lactobacillus/Clostridium* ratio following daily consumption of the test item suggests a higher intestinal population of *Lactobacillus*, supporting the survival of this lactic acid bacterium through the gastrointestinal tract. Similarly, Carnicer et al. also showed that the consumption of fermented milk containing *Lactobacillus casei* and *Streptococcus thermophilus* led to a reduction after 6 weeks in gram-negative aerobic flora in the treatment group, along with evidence of *L. casei* survival in the gastrointestinal tract [40].

The linear relationship observed between the change in the *Clostridium/E. coli* ratio (*Y*-axis) and serum IgG3 levels (*X*-axis) following probiotic supplementation indicates a significant association (Figure 1B). This suggests that approximately 62.4% of the variability in the *Clostridium/E. coli* ratio can be explained by variations in IgG3 levels, highlighting a strong correlation. Increases in serum IgG3 levels are associated with proportional increases in the *Clostridium/E. coli* ratio. The modulation of this ratio by probiotic supplementation suggests that the probiotic might influence both the gut microbiota composition and systemic immune responses. *Clostridium* species, particularly those within the *Clostridia* class, are often associated with beneficial metabolic and immunological functions, such as butyrate production and regulatory T cell induction [41,42]. In contrast, *E. coli* is frequently linked to dysbiosis and inflammatory conditions when present in elevated proportions. Thus, the observed relationship indicates that probiotic supplementation could promote microbiota changes that align with enhanced immune regulation and reduced inflammatory potential, potentially mediated by IgG3 activity [43]. FitzGerald et al. [44] conducted a randomized, double-blind, controlled trial to investigate the impact of a multi-strain probiotic (*L. paracasei* CNCM I-1518, CNCM I-3689, *L. rhamnosus* CNCM I-3690, and four yogurt strains) on gut microbiota recovery following a 14-day *Helicobacter pylori* eradication therapy. Metagenomic analysis revealed that ingested strains were transiently detected and replicated in fecal samples during and after antibiotic administration. Probiotic consumption led to a modest but significant improvement in microbiota recovery, with greater *L. paracasei* and *L. rhamnosus* abundance and an increased replication rate of *L. paracasei* CNCM I-1518 in individuals with better recovery. These findings provide mechanistic insights into probiotic-mediated microbiome protection during antibiotic treatment.

On the other hand, the relationship between the change in the *Lactobacillus/Clostridium* ratio (*Y*-axis) and the *Clostridium/E. coli* ratio (*X*-axis) following probiotic supplementation reveals a significant linear association (Figure 1C). This indicates that 56% of the variability in the *Lactobacillus/Clostridium* ratio can be explained by changes in the *Clostridium/E. coli* ratio, indicating a moderately strong negative correlation. As the *Clostridium/E. coli* ratio increases, the *Lactobacillus/Clostridium* ratio decreases. This inverse relationship reflects complex interactions within the gut microbiota during probiotic supplementation. This interplay might be attributed to competitive dynamics, differing ecological niches, or probiotic-induced changes in the gut environment, such as alterations in pH or nutrient availability [45,46]. The findings have important implications for understanding how probiotic supplementation modulates gut microbiota dynamics. *Lactobacillus* species are widely recognized for their beneficial roles in gut health, including the maintenance of intestinal barrier integrity and immune modulation.

Additionally, the modification observed in the *Lactobacillus/Clostridium* ratio, along with its correlation with other immunological biomarkers, reinforces the idea that these indicators could serve as early biomarkers to evaluate the impact of probiotic interventions on gut microbiota and their relationship with immune health. This approach aligns with recent studies proposing microbiota profiles as diagnostic and prognostic tools in the context of immunonutrition [47,48].

## 5. Conclusions

This study suggests that short-term (15-day) consumption of *L. paracasei* CNCM I-1518 could have effects on both the immune system and the gut microbiota composition.

The findings indicate that cellular immunity is enhanced through an increase in circulating B lymphocytes, higher absolute monocyte counts—potentially improving phagocytosis and antigen presentation—and an overall rise in leukocyte numbers. Additionally, a reduction in eosinophil levels was observed, which could help mitigate allergic responses. Humoral immunity was also positively impacted, based on the elevated serum levels of IgG1, IgG2, and IgG4, reflecting potentially improved immunological defenses against bacterial antigens. Furthermore, the serum levels of complement proteins C3 and C4, essential components of innate immunity for pathogen elimination and inflammation induction, were significantly increased.

In terms of gut microbiota modulation, the intervention resulted in a reduction in *Clostridium* levels and a lower *Clostridium/Escherichia coli* ratio. Most notably, the *Lactobacillus/Clostridium* ratio increased, emphasizing the strain’s potential ability to promote a more favorable intestinal bacterial balance.

However, the limitations of this study cover some aspects of the design and methodology. Its small sample size, with only 21 participants, may limit the generalizability of the findings to larger populations. Also, this study’s gender imbalance may limit the generalizability of results, as hormonal differences can affect gut microbiota and gut–brain axis responses. Future research should include a more balanced gender representation.

While the participants could be considered as their own control group at the baseline, the absence of a separate, independent control group and the lack of a double-blind design reduce the robustness of the evidence. Since no placebo was used during the intervention phases, this limits the ability to attribute observed effects solely to the intervention.

Additionally, the study did not include a wash-out period between interventions, which may have influenced the outcomes by not allowing for a complete clearance of the previous intervention’s effects. The relatively short duration of each intervention (15 days) may also not have been sufficient to capture long-term effects of the observed changes in immune function. Future studies with larger sample sizes, double-blind, placebo-controlled designs, longer interventions, and wash-out periods would be necessary to establish more robust scientific evidence. No a priori statistical power analysis was conducted, which limits the ability to determine whether the sample size was sufficient to detect meaningful differences. Future studies should ensure adequate power through proper sample size calculation based on expected effect sizes. On the methodology, the main limitations are that gut microbiota analyses provide only a relative, rather than absolute, count of specific microorganisms. This approach may miss a broader range of microbial diversity, as it focuses on only certain species.

Nonetheless, these preliminary results suggested the immunological and microbiota-modulating potential of *L. paracasei* CNCM I-1518, reinforcing its promise as a dietary intervention to support immune health. Future studies could build upon these findings to explore its effects over longer intervention periods and in different populations.

## Figures and Tables

**Table 1:** Lymphocyte subpopulations analyzed during the study.

Parameter	Extraction 1 (n = 19)	Extraction 2 (n = 21)	Extraction 3 (n = 21)
% lymphocytes T CD3+	73.55 ± 5.57 (63.59–80.55)	74.81 ± 6.04 (60.45–84.85)	74.75 ± 5.74 (60.1–82.35)
% lymphocytes T helper CD3+ CD4+	46.05 ± 5.87 (36.53–54.97)	46.15 ± 5.68 (36.10–57.20)	46.33 ± 4.59 (38.10–53.50)
% lymphocytes cytotoxic T CD3+ CD8+	25.04 ± 4.88 (16.01–34.10)	24.95 ± 3.77 (16.70–31.80)	24.86 ± 4.17 (16.50–30.90)
CD4/CD8 T cell ratio (T helper cells/cytotoxic T cells ratio).	1.93 ± 0.55 (1.21–2.98)	1.91 ± 0.47 (1.27–3.43)	1.92 ± 0.42 (1.43–3.22)
% lymphocytes T CD3+ CD4+ CD8+	4.63 ± 1.38 (2.01–7.05) ^1^	0.94 ± 0.44 (0.40–2.01) ^2^	0.65 ± 0.48 (0.16–2.47) ^3^
% lymphocytes T CD3+ CD4− CD8−	7.30 ± 3.11 (3.50–16.10) ^4^	4.29 ± 2.21 (1.64–9.53) ^5^	5.49 ± 4.06 (1.62–15.94)
% lymphocytes T CD3− CD8+	7.38 ± 2.97 (2.99–12.64) ^6^	5.87 ± 3.01 (2.20–11.70) ^7^	4.94 ± 2.22 (2.00–0.60)
% lymphocytes B CD19+	9.52 ± 2.97 (5.30–15.41) ^8^	11.27 ± 3.75 (5.60–21.30) ^9^	11.87 ± 4.39 (4.60–23.70)
Ratio of T/B CD3+/CD19+ lymphocytes	8.55 ± 2.96 (4.13–14.95) ^10^	7.35 ± 2.50 (2.95–14.08) ^11^	7.25 ± 3.10 (2.59–16.97)
% lymphocytes NK	16.75 ± 6.00 (7.05–27.22) ^12^	13.29 ± 6.64 (4.20–31.70) ^13^	12.85 ± 5.65 (6.70–33.40)
% lymphocytes CD3+ CD56+	7.84 ± 5.20 (0.97–22.08)	6.14 ± 3.52 (1.80–16.20) ^14^	5.28 ± 3.80 (1.40–18.50)
% naïve T lymphocytes	39.11 ± 17.85 (3.03–86.83) ^15^	49.14 ± 14.86 (19.48–90.25)	45.16 ± 15.40 (25.67–87.90) ^16^
Naïve T lymphocytes mean intensity of fluorescence (MFI)	51.54 ± 29.13 (33.22–165.93)	47.98 ± 9.83 (34.01–66.50)	39.52 ± 6.30 (26.32–50.90) ^17^
% T memory lymphocytes	42.19 ± 9.18 (27.89–57.69) ^18^	44.28 ± 8.48 (32.70–62.70)	41.08 ± 6.71 (27.35–52.41) ^19^
T memory lymphocytes mean intensity of fluorescence (MFI)	84.21 ± 35.89 (46.84–185.52) ^20^	130.50 ± 29.70 (87.92–197.30)	96.51 ± 19.33 (66.39–141.18) ^21^
% Naïve helper T lymphocytes	25.39 ± 10.12 (11.23–55.20)	25.56 ± 8.79 (8.77–45.14) ^22^	21.05 ± 8.52 (7.67–45.51) ^23^
Naïve helper T lymphocytes mean intensity of fluorescence (MFI)	38.05 ± 6.75 (26.96–52. 16)	40.34 ± 8.32 (27.84–56.18) ^24^	34.92 ± 5.34 (24.82–46.15) ^25^
% Memory helper T lymphocytes	30.50 ± 6.54 (18.41–46.54)	30.27 ± 5.83 (20.56–40.41)	28.41 ± 5.15 (18.50–38.69) ^26^
Memory helper T lymphocytes mean intensity of fluorescence (MFI)	92.50 ± 44.82 (51.09–217.50) ^27^	146.17 ± 28.07 (110.67–212.87) ^28^	112.29 ± 20.39 (83.91–159.43) ^29^
% Naïve cytotoxic T lymphocytes	23.64 ± 8.71 (10.77–48.39) ^30^	21.13 ± 6.14 (10.80–38.34) ^31^	19.35 ± 6.55 (6.73–36.83) ^32^
Naïve cytotoxic T lymphocytes mean intensity of fluorescence (MFI)	50.75 ± 15.11 (36.43–84.82)	58.67 ± 14.24 (37.52–81.03)	44.21 ± 7.04 (26.63–53.85) ^33^
% Memory cytotoxic T lymphocytes	16.61 ± 5.05 (4.44–26.72) ^34^	12.78 ± 3.77 (6.67–21.35) ^35^	11.18 ± 3.30 (4.99–17.03) ^36^
Memory cytotoxic T lymphocytes mean memory cytotoxic T lymphocytes	77.91 ± 28.52 (43.06–159.68) ^37^	101.46 ± 26.91 (65.44–163.89)	76.73 ± 14.99 (52.71–110.21) ^38^
Ratio of naïve T lymphocytes to memory	1.04 ± 0.67 (0.07–2.76)	1.19 ± 0.49 (0.32–2.26)	1.17 ± 0.54 (0.49–2.24)
Ratio of naïve helper T cells to memory	0.91 ± 0.54 (0.34–2.38)	0.92 ± 0.45 (0.23–2.11)	0.80 ± 0.43 (0.26–1.98) ^39^
Ratio of naïve cytotoxic T lymphocytes to memory	1.76 ± 1.50 (0.64–7.12)	1.81 ± 0.79 (0.77–4.06)	1.95 ± 1.03 (0.58–4.32)

^1^ *p* value = 9 × 10^−10^ (1 vs. 2); ^2^ *p* value = 3 × 10^−10^ (1 vs. 3); ^3^ *p* value = 0.002 (2 vs. 3); ^4^ *p* value = 9.02 × 10^−8^ (1 vs. 2); ^5^ *p* value = 0.030 (1 vs. 3); ^6^ *p* value = 0.004 (1 vs. 2); ^7^ *p* value = 0.0001 (1 vs. 3); ^8^ *p* value = 0.009 (1 vs. 2); ^9^ *p* value = 0.002 (1 vs. 3); ^10^ *p* value = 0.014 (1 vs. 2); ^11^ *p* value = 0.003 (1 vs. 3); ^12^ *p* value = 0.011 (1 vs. 2);^13^ *p* value = 0.004 (1 vs. 3); ^14^ *p* value = 0.0007 (1 vs. 3); ^15^ *p* value = 0.006 (1 vs. 2); ^16^ *p* value = 0.005 (2 vs. 3); ^17^ *p* value = 0.0002; ^18^ *p* value = 0.018 (1 vs. 2); ^19^ *p* value = 0.031 (2 vs. 3); ^20^ *p* value = 0.0003 (1 vs. 2); ^21^ *p* value = 0.0002 (2 vs. 3); ^22^ *p* value = 0.013 (1 vs. 3); ^23^ *p* value = 0.0001 (2 vs. 3); ^24^ *p* value = 0.035 (1 vs. 3); ^25^ *p* value = 0.0009 (2 vs. 3); ^26^ *p* value = 0.01 (2 vs. 3); ^27^ *p* value = 0.0007 (1 vs. 2); ^28^ *p* value = 0.03 (1 vs. 3); ^29^ *p* value = 0.00003 (2 vs. 3); ^30^ *p* value = 0.027 (1 vs. 2) ^31^ *p* value = 0.006 (1 vs. 3); ^32^ *p* value = 0.012 (2 vs. 3); ^33^ *p* value = 0.00003 (2 vs. 3); ^34^ *p* value = 0.00009 (1 vs. 2); ^35^ 0.000009 (1 vs. 3); ^36^ *p* value = 0.022 (2 vs. 3); ^37^ *p* value = 0.012 (1 vs. 2); ^38^ *p* value = 0.0005 (2 vs. 3); ^39^ *p* value = 0.003 (2 vs. 3).

**Table 2:** Serum immune proteins analyzed during the study.

Parameter (Unit)	Extraction 1 (n = 19)	Extraction 2 (n = 21)	Extraction 3 (n = 21)
C3 (mg/dL)	107.94 ± 16.02 (87.10–147.60) ^1^	94.78 ± 11.77 (77.60–123.20)	104.09 ± 15.84 (79.20–127.30) ^2^
C4 (mg/dL)	19.49 ± 3.94 (13.50–27.00) ^3^	16.40 ± 2.96 (11.30–23.90)	18.09 ± 5.38 (12.80–36.30) ^4^
IgG (mg/dL)	1196.08 ± 152.16 (898.00–1476.00)	1208.01 ± 181.95 (873.00–1630.00)	1174.96 ± 201.88 (736.00–1638.00)
IgA (mg/dL)	221.14 ± 61.01 (132.00–347.00)	210.99 ± 64.87 (109.00–340.90)	225.53 ± 67.39 (111.40–352.8)
IgM (mg/dL)	124.02 ± 35.82 (62.70–206.90)	131.78 ± 52.32 (62.20–298.10)	133.08 ± 49.30 (71.00–303.00)
IgG1 (mg/dL)	564.06 ± 102.94 (320.00–763.00) ^5^	521.82 ± 108.15 (300.10–697.10) ^6^	613.32 ± 145.11 (290.90–862.10) ^7^
IgG2 (mg/dL)	305.88 ± 80.21 (179.90–445.00)	305.37 ± 88.17 (145.60–495.30) ^8^	359.46 ± 106.35 (183.10–592.00) ^9^
IgG3 (mg/dL)	45.52 ± 18.21 (11.10–75.90) ^10^	45.93 ± 19.45 (13.70–91.10)	46.60 ± 19.86 (12.00–103.70)
IgG4 (mg/dL)	43.83 ± 17.20 (19.90–84.50) ^11^	39.15 ± 15.32 (15.70–73.20)	46.47 ± 19.31 (16.30–88.90) ^12^

^1^ *p* value = 0.00001 (1 vs. 2); ^2^ *p* value = 0.0005 (2 vs. 3); ^3^ *p* value = 0.0004 (1 vs. 2); ^4^ *p* value = 0.021 (2 vs. 3); ^5^ *p* value = 0.002 (1 vs. 2); ^6^ *p* value = 0.009 (1 vs. 3); ^7^ *p* value = 0.00001 (2 vs. 3); ^8^ *p* value = 0.0005 (1 vs. 3); ^9^ *p* value = 0.000004 (2 vs. 3); ^10^ *p* value = 0.024 (1 vs. 2); ^11^ *p* value = 0.00005 (1 vs. 2); ^12^ *p* value = 0.005 (2 vs. 3).

**Table 3:** Fecal flora analyses analyzed during the study.

Parameter (Unit)	Extraction 1 (n = 19)	Extraction 2 (n = 21)	Extraction 3 (n = 21)
*E. coli* (log)	6.41 ± 2.04 (4.00–10.00)	6.70 ± 1.66 (4.00–9.30)	6.60 ± 1.31 (4.00–8.43)
*Clostridium* (log)	10.83 ± 0.73 (9.89–12.00)	10.09 ± 1.05 (8.18–12.00) ^1^	9.98 ± 0.96 (8.40–12.00)
*Lactobacillus* (log)	7.05 ± 0.96 (5.85–9.25)	7.70 ± 0.74 (6.03–8.56)	7.41 ± 1.00 (5.86–9.18)
*Clostridium/E. coli*	4.42 ± 1.96 (1.34–7.68) ^2^	3.38 ± 1.42 (0.75–5.13)	3.65 ± 1.17 (2.10–6.05)
*Lactobacillus/E. coli*	0.64 ± 2.00 (−2.75–3.81)	1.00 ± 1.80 (−1.93–3.13)	0.81 ± 1.23 (−1.02–3.81)
*Lactobacillus/Clostridium*	−3.78 ± 1.02 (−5.16–−2.21) ^3^	−2.38 ± 1.29 (−4.35–0.04) ^4^	−2.39 ± 0.97 (−3.84–−1.05)

^1^ *p* value = 0.042 (1 vs. 3); ^2^ *p* value = 0.041 (1 vs. 2); ^3^ *p* value = 0.007 (1 vs. 2); ^4^ *p* value = 0.020 (1 vs. 3).

## Data Availability

The original contributions presented in this study are included in the article/Supplementary Material. Further inquiries can be directed to the corresponding author.

## Supplementary Materials

The following supporting information can be downloaded at: https://www.mdpi.com/article/10.3390/nu17142287/s1, Table S1: Biochemical parameters analysed during the study; Table S2: Haematology parameters analysed during the study; Table S3: Correlations between immunological and microbiota changes (Δ values).
