# Supplementary material for: Exploratory Study on Microbiota and Immune Responses to Short-Term L. paracasei CNCM I-1518 Consumption in Healthy Adults
Source: Nutrients. 2025 Jul 10;17(14):2287. doi: 10.3390/nu17142287 (PMC12300870; doi:10.3390/nu17142287)
Supplement: Supplementary file 1 [file nutrients-17-02287-s001.zip › nutrients-3728568-supplementary.pdf]

Supplementary material

Table S1: Biochemical parameters analysed during the study

	Extraction 1 (n=19)	Extraction 2 (n=21)	Extraction 3 (n=21)	Reference value
Urea (mg/dL)	28.95 ±7.27 (16.00-43.00)	29.48±7.24 (21.00-45.00)	29.86±6.67 (18.00-47.00)	10 - 50
Glucose (mg/dL)	86.84 ±5.38 (78.00 – 100.00)	85.00±6.71 (74.00-100.00)	85.62±7.36 (77.00-104.00)	60 - 110
Creatinine (mg/dL)	0.82 ±0.13 (0.60-1.10)	0.81±0.14 (0.60-1.10)	0.78±0.13 (0.60-1.10)[1]	0.5-1.2
Uric acid (mg/dL)	4.25 ±1.13 (2.30 – 6.80)	4.27 ± 1.03 (2.80 – 6.80)[2]	4.54 ± 1.11 (2.80 – 6.80)[3]	2.4-5.7
Triglycerides (mg/dL)	59.42 ±20.75 (35.00 – 122.00)	60.38 ± 18.11 (39.00 – 116.00)	67.95 ± 56.87 (25.00 – 284.00)	36-165
Total cholesterol (mg/dL)	167.89 ±33.07 (136.00-242.00)[4]	151.33 ± 26.98 (116.00 – 208.00)[5]	159.38 ± 31.61 (125.00 – 233.00)	120-220
Total proteins (g /dL)	7.83 ±0.30 (7.40 – 8.60)[6]	7.45 ± 0.38 (6.70 – 8.00)[7]	7.47 ± 0.39 (6.90 – 8.60)	6-8.4
Albumin (g/dL)	4.76 ± 0.21 (4.40 – 5.20)	4.70 ± 0.25 (4.20 – 5.10)	4.69 ± 0.21 (4.20 – 5.00)	3.5-5
Alkaline phosphatase (U/L)	67.32 ± 18.08 (48.00 – 120.00)	67.71 ± 16.67 (44.00 – 115. 00)	68.86 ± 19.72 (43.00 – 124.00)	35-104
Calcium (mg/dL)	9.75±0.23 (9.20-10.20)	9.68±0.28 (9.10-10.30)[8]	9.50±0.20 (9.10-9.70) [9]	8-11
Inorganic phosphorus (mg/dL)	3.85 ±0.37 (3.00-4.40)	3.81 ±0.44 (2.80-4.50)	3.96 ±0.45 (3.20-4.70)	2.7-4.5

Iron (ug/dL)	104.58 ±54.85 (21.00-260.00)	90.00±29.96 (33.00-137.00)[10]	68.38±33.36 (26.00-144.00) [11]	50-150
Sodium (mEq/L)	138.26 ±1.66 (135.00-141.00)	139.19 ±1.78 (135.00-142.00)	137.57 ±2.04 (133.00-142.00)[12]	135-153
Potassium (mEq/L)	4.21 ± 0.25 (4.00-5.00)	4.15 ±0.29 (3.60 – 4.80)	4.17 ±0.32 (3.50 – 5.00)	3.5-5.3
Chloride (mEq/L)	103.89 ±2.51 (98.00 – 106.00)[13]	105.33 ± 2.58 (101.00 – 109.00) [14]	108.33 ± 2.75 (102.00 – 110.00)	90-110
Transferrin (mg/dL)	255.95 ±37.24 (204.00 – 344. 00)[15]	241.62 ±37.33 (199.00 – 374.00) [16]	246.52 ±34.36 (205.00-331.00)	200-360

[1] p value = 0.042 between extraction 2 and 3 (2 vs 3); [2] p value = 0.0003 (1 vs 3); [3] p value = 0.004 (2 vs 3); [4] p value = 0.0009 (1 vs 2); [5] p value = 0.033 (1 vs 3): [6] p value = 0,000003 (1 vs 2); [7] p value = 0.00002 (1 vs 3); [8] p value =0,00009 (1 vs 3): [9] p value = 0.008 (2 vs 3); [10] pvalue= 0,013 (1 vs 3); [11] pvalue= 0,018 (2 vs 3); [12] p value = 0.003 (2 vs 3); [13] p value = 0,047 (1 vs2); [14] p value = 0,003 (1 vs 3); [15] p value = 0.007 (1 vs 2); [16] pvalue = 0.044 (1 vs 3)

Table S2: Haematology parameters analysed during the study

**Parameter**	**Extraction 1 (n=19)**	**Extraction 2 (n=21)**	**Extraction 3 (n=21)**	**Reference value**
Erythrocytes (10^6 /uL)	4.80 ±0.31 (4.25 – 5.58) [1]	4.49 ±0.33 (3.88 - 5.31) [2]	4.50 ±0.35 (4.00 - 5.33)	4.2-5.2
Haemoglobin (g/dL)	14.45±0.80 (13.40 – 16.30)	14.21±0.93 (12.80 – 16.10) [3]	13.97±0.93 (12.70 – 16. 10) [4]	12-16
Haematocrit (%)	43.34 ±2.06 (40.00 – 48. 00)[5]	40.82 ±2.70 (36.00 – 46.00) [6]	41.94 ±2.93 (37.00 – 48.00) [7]	36-46

Mean Corpuscular Volume (MCV) (fL)	90.41 ±3.37 (84.10 – 95.80) [8]	91.05 ±3.39 (85.30 – 96.90) [9]	93.36 ±4.26 (87.20 – 100.60) [10]	80-99
Mean Corpuscular Hemoglobin (MCH) (pg)	30.12 ±1.24 (27.50 – 32.10) [11]	31.71 ±1.29 (29.00 – 34.00) [12]	31.07 ±1.19 (29.20- 33.40) [13]	26-32
Mean Corpuscular Haemoglobin Concentration (MCHC) (%)	32.30 ±4.44 (14.10 – 34.50) [14]	34.81 ±0.60 (33.40 – 35.70)	33.29 ±0.54 (32.50-34.50) [15]	33-37
RDW % (Red Cell Distribution Width)	13.83 ±0.63 (12.90 – 15.60) [16]	13.63 ±0.66 (13.00 – 15.00) [17]	14.57 ±0.72 (13.50-16.50) [18]	11.5-14.5
Leukocytes, White Blood Cells (WBC) (10^3/µl)	6.69 ±1.14 (4.84 – 9.50)	6.91 ±1.85 (4.27 – 11.68) [19]	7.67 ±2.10 (3.84 – 11.97)	4.5-10
Lymphocytes (%)	35.91 ±9.38 (18.00 – 64.00)	34.00 ±7.54 (21.10 – 49.00)	32.86 ±7.89 (17.70-45.90)	20-51
Absolute Lymphocyte Count (10^3/µl)	2.29 ±0.53 (0.91 – 3.11)	2.29 ±0.57 (1.31 – 3.38)	2.41 ±0.51 (1.66 – 3.50)	0.9-5.2
Monocytes (%)	7.42 ±8.26 (2.00 – 41.00)	5.13 ±1.23 (3.00- 8.00)	5.32 ±1.46 (2.70-8.00)	1.3-9.3
Absolute Monocytes Count (10^3/µl)	0.38 ±0.08 (0.27 – 0.57)	0.34 ±0.70 (0.21- 0.48)	0.40 ±0.11 (0.15 – 0.57) [20]	0.16-1
Neutrophils (%)	51.37 ±10.00 (22.00-73.10)	54.48 ±9.36 (39.10-71.50)	55.61 ±9.06 (43.70-75.90)	42-76
Absolute Neutrophils Count	3.54 ±0.79 (2.48 – 5.36)	3.86 ±1.60 (1.92 – 7.62)	4.41 ±1.82 (1.83 – 9.09)	1.9-8

Eosinophils (%)	3.87 ±2.46 (0.30 – 10.00)	4.00 ±3.04 (0.50 – 11.30)	3.10 ±1.85 (0.90 – 7.60)	0-5
Basophils (%)	0.75 ±0.41 (0.00 – 1.80)	0.69 ± 0.43 (0.30 – 1.60)	0.71 ± 0.32 (0.30 – 1.50)	0-2
Platelets (10^3/µL)	231.95 ± 42.67 (160.00 – 292.00)	230.29 ± 44.34 (158.00 – 328.00)	243.14 ± 49.99 (132.00 – 323.00)	150-400
Mean Platelet Volume (MPV) (fL)	9.37 ±1.06 (8.10- 11.80)	9.53 ±0.98 (8.30 – 11.90)	9.23±1.02 (7.90 – 12.00) [21]	7.2-11.1

[1] p value = 9,7E-10 (1 vs 2); [2] p value = 2E-8 (1 vs 3); [3] p value = 0.0003 (1 vs 3); [4] p value = 0.034 (2 vs 3); [5] p value = 5E-9 (1 vs 2); [6] p value = 0.005 (1 vs 3): [7] p value = 0.006 (2 vs 3); [8] p value = 8E-7 (1 vs 2); [9] p value = 2E-10 (1 vs 3); [10] p value = 8E-8 (2 vs 3); [11] p value =2E-11(1 vs 2); [12] p value =1E-10 (1 vs 3); [13] p value = 1E-06 (2 vs 3); [14] p value = 0,0243 (1 vs 2); [15] p value = 2E-09 (2 vs 3); [16] p value = 0.027 (1 vs 2); [17] p value = 3E-07 (1 vs 3); [18] p value = 1E-10 (2 vs 3); [19] p value = 0.043 (1 vs 3); [20] p value = 0.0096 (2 vs 3); [21]p value= 0,001 (2 vs 3).

Table S3. Correlations between immunological and microbiota changes (Δ values).

**Variable 1**	**Variable 2**	**Type of regression**	**R square**	**P value**
**(LAB supplementation – basal) Δ values**				
T/B ratio	% B cells	Linear -	0.543	<0.0001
% $CD3^-CD8^+$ cells	% B cells	Linear -	0.508	<0.001
% naive Th cells	% total naive T cells	Linear +	0.665	<0.0001
MFI memory Tc cells	MFI total memory T cells	Linear +	0.984	<0.0001
Naive/memory ratio in Th cells	% of naive Th cells	Linear +	0796	<0.0001
MFI naive T cells	% naive T cells	Cubic	0.959	<0.0001
IgG1 in serum	% memory Tc cells	Cubic	0.621	0.002
IgG4 in serum	% memory Tc cells	Cubic	0.686	<0.0001
Foecal Clostridium/E.Coli ratio	% $CD3^-CD8^+$ cells	Cubic	0.675	0.034
Foecal Clostridium/E.Coli log ratio	IgG3 in serum	Linear +	0.624	0.011
Foecal Lactobacillus/Clostridium log ratio	Foecal Clostridium/E.Coli log ratio	Linear -	0.560	0.020
Foecal Clostridium	% $CD3^+$ $CD4^+CD8^+$ cells	Cubic	0.802	0.033

Foecal Clostridium/E.Coli ratio	MFI total memory T cells	Linear +	0.554	0.021
IgG4 in serum	IgG3 in serum	Cubic	0.737	<0.0001
IgG4 in serum	IgG2 in serum	Cubic	0.496	0.014
IgG4 in serum	IgG1 in serum	Cubic	0.548	0.007
C3 in serum	C4 in serum	Linear +	0.584	<0.0001
C4 in serum	Naive/memory Th ratio	Cubic	0.594	0.003
Foecal Clostridium log	% $CD3^-CD8^+$ cells	Quadratic	0.649	0.043
Foecal Clostridium log	MFI memory Tc cells	Quadratic	0.861	0.003
Foecal Lactobacillus/Clostridium ratio	MFI naive Tc cells	Quadratic	0.781	0.011
**(LAB deprivation – basal) Δ values**				
MFI memory T cells	MFI memory Tc cells	Linear +	0.967	<0.0001
% naive T cells	MFI naive Tc cells	Linear +	0.529	<0.0001
MFI naive Th cells	MFI memory Tc cells	Linear +	0.649	<0.0001
MFI memory Th cells	MFI memory Tc cells	Linear +	0.767	<0.0001
MFI naive Tc cells	MFI memory Tc cells	Linear +	0.526	<0.0001
MFI naive Th cells	MFI memory Th cells	Linear +	0.586	<0.0001
IgG2 in serum	Foecal Clostridium/E.Coli ratio	Cubic	0.790	0.004
C3 in serum	C4 in serum	Linear +	0.624	<0.0001
**(LAB supplementation – LAB deprivation) Δ values**				
MFI total memory T cells	MFI memory Tc cells	Linear +	0.917	<0.0001
MFI memory Th cells	MFI memory Tc cells	Linear +	0.568	<0.0001
MFI naive Tc cells	MFI memory Tc cells	Linear +	0.646	<0.0001
MFI naive Th cells	MFI memory Th cells	Linear +	0.707	<0.0001
Foecal Clostridium/E.Coli log ratio	Foecal Clostridium log	Linear +	0.751	0.003
Foecal Clostridium	Foecal Lactobacillus/Clostridium ratio	Linear -	0.520	0.028
C3 in serum	C4 in serum	Linear +	0.432	0.001

Tc: cytotoxic T cells; Th: helper T cells; MFI: Mean Fluorescence Index
